# Evaluating the technical feasibility of biology-guided dose painting in proton therapy

**DOI:** 10.1016/j.phro.2025.100832

**Published:** 2025-08-27

**Authors:** Caterina Brighi, Giovanni Parrella, Letizia Morelli, Silvia Molinelli, Giuseppe Magro, Sara Lillo, Alberto Iannalfi, Mario Ciocca, Sara Imparato, David E.J. Waddington, Paul Keall, Chiara Paganelli, Ester Orlandi, Guido Baroni

**Affiliations:** a Department of Electronics, Information and Bioengineering, Politecnico di Milano, Milan, Italy; b Image X Institute, Sydney School of Health Sciences, Faculty of Medicine & Health, The University of Sydney, Sydney, Australia; c National Center for Oncological Hadrontherapy, CNAO, Pavia, Italy; d Department of Internal Medicine and Therapeutics, University of Pavia, Pavia, Italy; e Department of Clinical, Surgical, Diagnostic, and Pediatric Sciences, University of Pavia, Pavia, Italy

**Keywords:** Dose painting, Proton therapy, Functional MRI, Pencil beam scanning, Skull-base chordoma, Planning study

## Abstract

Biology-guided voxel-level inverse prescription mapping for dose painting (DP) using diffusion-weighted magnetic resonance imaging was evaluated for technical feasibility in proton therapy for 10 skull-base chordoma patients. Patient-specific DP prescriptions were generated from tumour cellularity and implemented in a clinical treatment planning system. Compared with uniform plans, DP achieved lower conformity (although >97 %), improved target dose metrics, reduced doses to most organs at risk, and increased tumour control probability without exceeding clinical constraints. DP proton therapy is technically feasible and may enhance treatment effectiveness.

## Highlights

• Voxel-level inverse prescription mapping for dose painting in skull-base chordoma.
• Dose painting proton plans achieved over 97% voxel-level conformity.
• Median tumour dose rose to 75.1 Gy and maximum reached 78.2 Gy with dose painting.
• Tumour control probability improved from 0.09 to 0.14 with dose painting.
• Dose constraints to all organs at risk were met using dose painting in 10 patients.

## Graphical abstract

**Figure:**
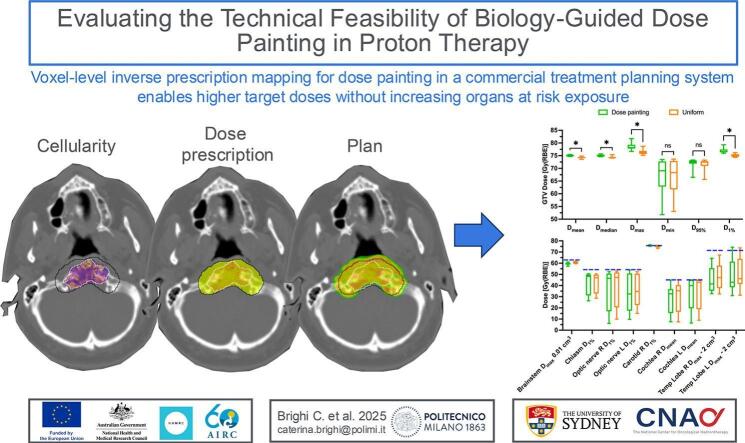

## 1 Introduction

Dose painting (DP) is a radiotherapy strategy that delivers spatially heterogeneous doses within the tumour, aiming to escalate radiation to sub-volumes at higher risk of recurrence while respecting organ-at-risk (OAR) constraints. Functional imaging, such as positron emission tomography (PET) or advanced magnetic resonance imaging (MRI), can characterise tumour biology—including cellularity, hypoxia, and metabolism—providing spatial information to guide DP [1,2].

While numerous studies have implemented DP in photon therapy using intensity-modulated radiation therapy (IMRT) or volumetric modulated arc therapy (VMAT) [[3], [4], [5], [6], [7], [8], [9], [10], [11], [12], [13], [14], [15], [16], [17], [18]], its application in particle therapy, particularly proton therapy with pencil beam scanning (PBS), remains underexplored [[19], [20], [21]]. Proton therapy offers dosimetric advantages due to the Bragg peak, but current treatment planning systems (TPS) have more limited dose modulation capabilities than photon-based platforms, posing challenges for fine-grained biological dose escalation. Demonstrating the technical feasibility of DP in a clinical proton TPS is therefore an important step toward future clinical adoption.

Proton and light ion therapy are established treatments for skull-base chordomas (SBC) and chondrosarcomas, achieving high-dose delivery to the tumour, while sparing nearby critical structures such as the brainstem, optic nerves, and temporal lobes [[22], [23], [24], [25], [26], [27], [28], [29], [30], [31]]. However, standard practice generally applies uniform dose escalation to the entire target volume, regardless of biological heterogeneity. A biologically informed approach using functional imaging could allow selective dose boosting to radioresistant subregions, potentially exceeding uniform boost levels while maintaining OAR safety.

In this study, we investigated the feasibility of diffusion-weighted MRI (DWI)-guided DP in proton therapy for SBC patients. Patient-specific DP prescriptions were derived from tumour cellularity maps and implemented in a clinical PBS TPS. We compared DP and uniform prescription plans in terms of dose conformity, target coverage, and OAR sparing. Additionally, we estimated tumour control probability (TCP) to assess whether biology-guided DP could provide potential clinical benefit over standard uniform prescriptions.

## 2 Materials and methods

### 2.1 Patient dataset

This retrospective study analysed pre-treatment MRI and radiotherapy planning data from 10 SBC patients treated with proton therapy at the National Center for Oncological Hadrontherapy (CNAO) between 2013 and 2017, within the AIRC project (Ref. 24946; CNAO ethics approval CNAO 43 2021). Detailed MRI acquisition protocols, image preprocessing, target delineation steps, and patient characteristics are provided in Supplementary A and Table S1.

### 2.2 Dose painting prescriptions

DWI-derived cellular density values within the gross tumour volume (GTV) were multiplied by voxel volume to estimate the number of cells per voxel, $\rho_{i}$. Assuming that higher cellularity corresponds to higher tumour aggressiveness [32], a linear scaling function (Eq. (1)) was used to map cell number to dose prescriptions per voxel within the GTV. This approach was chosen for its simplicity, reproducibility and consistency with previous exploratory studies on functional image-guided DP [4].

(1) $$D_{\mathrm{presc},i}=\frac{\left(D_{\max}-D_{\min}\right)∙\left(\rho_{i}-\rho_{\min}\right)}{\rho_{\max}-\rho_{\min}}+D_{\min}$$

$D_{\min}$, minimum prescribed dose corresponding to the dose delivered to the GTV as per standard of care (74 Gy(RBE)^1^); $\rho_{i}$, number of cells per voxel; $\rho_{\max}$ and $\rho_{\min}$, maximum and minimum voxel cell counts in the GTV; $D_{\max}$, maximum prescribed dose corresponding to 110 % of the original dose delivered to the GTV (81 Gy(RBE)). Voxels in the safety margin region between GTV and clinical target volume (CTV) received at least the minimum dose prescription of 74 Gy(RBE). Dose prescription maps were obtained in CT image space by applying the inverse of the CT registration transformation matrix to DWI-MRI images. For DP planning, DP prescription maps within the CTV were converted into an inverse dose prescription, $D_{\mathrm{inv},i}$ according to equation (2):

(2) $$D_{\mathrm{inv},i}=D_{\max}-D_{\mathrm{presc},i}$$

The inverse dose prescription map was then imported into the TPS. The code developed for image processing is available on GitHub: https://doi.org/10.5281/zenodo.14930761.

### 2.3 Treatment planning

#### 2.3.1 Dose painting plans

Plans were generated in RayStation 2023B (RaySearch Laboratories) with a PBS delivery system and robust optimisation. The inverse dose map was used as background beamset dose, and optimisation proceeded in dose summation mode [10]. A constant RBE of 1.1 was applied for proton RBE-weighted dose calculation. All patients were planned with four fields, a 3 mm scan step, 2 mm energy slice spacing, and 37 fractions. Objectives and constraints are shown in Table 1. Additional details on optimisation and quality assessment, and clinical goals for the targets and OARs are in Supplementary B and Table S2.

**Table 1:** Objectives and constraints set for radiotherapy targets and organs at risk for plan optimisation.

Region of interest	Dose painting plans	Uniform plans
	*Objectives*	
GTV	min DVH of 74 Gy(RBE) to 95 % volume	
CTV	min DVH of 74 Gy(RBE) to 95 % volume*	
CTV	uniform dose 81 Gy(RBE)	uniform dose 74 Gy(RBE)
Brainstem	max dose of 61 Gy(RBE)*	
Chiasm	max DVH 54 Gy(RBE) to 1 % volume*	
Optic nerves	max DVH 50 Gy(RBE) to 1 % volume	
Cochleae	max EUD 45 Gy(RBE) parameter a = 1	
Carotids	max DVH 75.5 Gy(RBE) to 1 % volume	
Temporal lobes	max DVH 71 Gy(RBE) to 1.7 % volume	

	*Constraints*	
CTV	max dose 83 Gy(RBE)	max dose 76 Gy(RBE)
Brainstem	max dose 61 Gy(RBE)	

* Robust.

#### 2.3.2 Uniform prescription plans

To ensure consistency in optimisation algorithm, the uniform plans were created on the same TPS using identical beam geometry, delivery parameters, and optimisation settings as for DP plans, with the only difference being a uniform target dose prescription (Tables 1 and S2). Additional details on the plans’ comparison strategy are in Supplementary B. Robust evaluation of the uniform plans, including potential iterative re-optimisation to achieve clinical approval by an experienced medical physicist, was performed as per the DP plans.

### 2.4 Plan conformity

The conformity of the plans to their dose prescriptions in the GTV and CTV was evaluated with a quality factor (QF), as previously defined [3]:

(3) $$\mathrm{QF}=100-\frac{1}{n}\sum_{i}\left|\frac{{\mathrm{Dose}}_{\mathrm{plan},i}-{\mathrm{Dose}}_{\mathrm{presc},i}}{{\mathrm{Dose}}_{\mathrm{presc},i}}\right|$$

n, number of voxels within the target; ${\mathrm{Dose}}_{\mathrm{plan},i}$, dose planned for voxel i; ${\mathrm{Dose}}_{\mathrm{presc},i}$, dose prescribed for voxel i. A QF close to 100 % corresponds to an ideal plan, where the delivered dose perfectly matches the per-voxel dose prescription. The treatment planning goal for good conformity of DP plans was QF ≥95 %, as previously done for DP photon plans in head and neck cancer [12]. Per-voxel QF maps were generated to visualise where the plans deviated from the prescriptions in the target volumes.

### 2.5 Dose metrics

Mean (D_mean_), median (D_median_), maximum (D_max_), minimum dose (D_min_), dose delivered to 95 % (D_95%_) and 1 % of the volume (D_1%_) were calculated in the GTV and CTV. Dose metrics calculated for OARs corresponded to the clinical goals (Table S2). Equivalent uniform doses (EUD) for OARs were also estimated according to the method proposed in [33].

### 2.6 Tumour control probability

TCP in the GTV was calculated for both the DP and the uniform plans according to the Poissonian TCP LQ model, as per Eq. (4):

(4) $$\mathrm{TCP}=\prod_{i=1}^{Z}e^{-{N0}_{i}e^{\left(-\alpha D_{i}-\frac{D_{i}^{2}\beta }{n}\right)}}$$

${N0}_{i}$, number of cells per voxel calculated from the patient-specific DWI data; α and β, photon radiosensitivity parameters; $D_{i}$, planned RBE dose per voxel; n, number of fractions (i.e. 37); Z, total number of voxels in the target. $\alpha =0.1{\mathrm{Gy}}^{-1}$ as reported by Kamp et al. [34] and $\frac{\alpha }{\beta }=2.4Gy$ as reported by Paganetti [35], who performed survival experiments on chordoma cell lines.

### 2.7 Statistical analysis

Statistical analysis was performed in GraphPad Prism 10.4.0. Non-parametric, Wilcoxon matched-pairs signed-rank tests with Holm–Sidak correction were used to compare QF, dose metrics, and TCP between DP and uniform plans. Significance was set at p < 0.05.

## 3 Results

### 3.1 Plan conformity

Both DP and uniform plans met clinical acceptability, with QF values > 97 % in all patients (Tables S3 and S4). DP plans showed slightly lower conformity than uniform plans in both GTV (median 98.5 vs 99.2, p = 0.004) and CTV (98.7 vs 99.2, p = 0.004) (Figs. S1 and S2a, Tables S3 and S4).

### 3.2 Dose metrics

**Fig. 1:**
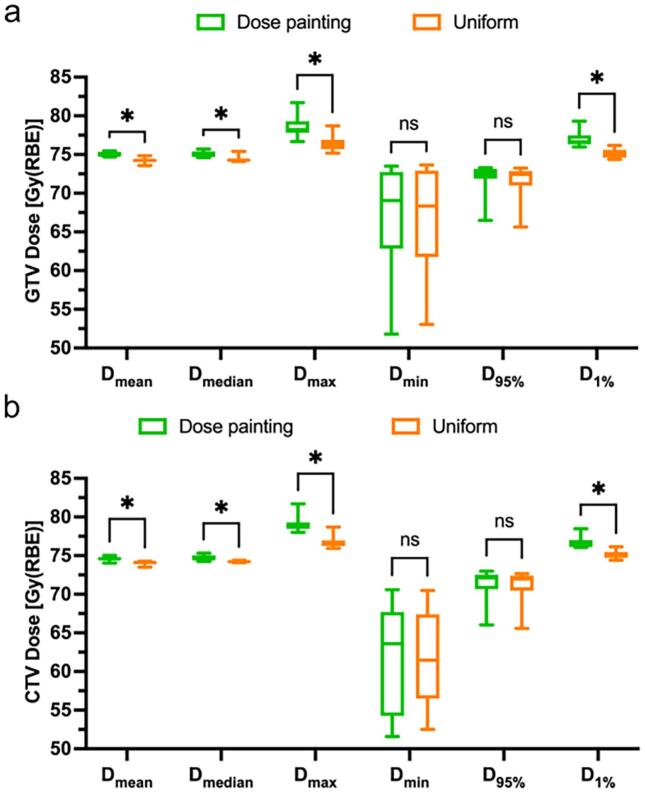
Dose metrics to radiotherapy targets. Box and whiskers plots of comparison of dose metrics in the **a**) GTV and **b**) CTV between dose painting and uniform plans. The bar in the boxes indicates the median value. CTV, clinical target volume; D_mean_, mean dose; D_median_, median dose; D_max_, maximum dose; D_min_, minimum dose; D_95%_, dose to 95 % of volume; D_1%_, dose to 1 % of volume; GTV, gross tumour volume. *p < 0.05, ns = p > 0.05. N = 10.

DP increased D_mean_, D_median_, D_max_, and D_1%_ in both GTV and CTV compared with uniform plans (p < 0.05) (Fig. 1, Tables S5 and S6). D_min_ and D_95%_ were also higher, though differences were not significant (p > 0.05). Dose–volume histograms confirmed these trends (Fig. S3).

All OAR constraints were met for all patients in both planning strategies. DP plans generally reduced dose metrics and equivalent uniform dose to most OARs, though no differences reached statistical significance (Figs. S4 and S5**,** Tables S7, S8 and S9). Dose–volume histograms confirmed these trends (Fig. S6).

### 3.3 Tumour control probability

TCP in the GTV was significantly higher for DP than uniform plans (median 0.14 vs 0.09, p = 0.004) (Fig. S2b, Tables S3 and S4).

## 4 Discussion

This study evaluated the technical feasibility and potential benefits of biologically guided DP proton plans using a clinical TPS in 10 SBC patients, compared with uniform proton plans. Although DP plans showed slightly lower conformity than uniform plans, the QF in target volumes exceeded 97 %, above the 95 % threshold for good conformity. Reduced conformity primarily arose in the dose-modulated regions: median QF values were similar in the GTV and CTV, with slightly higher variability in the GTV. Occasional low-QF regions extended beyond the GTV (Fig. S1), suggesting that even limited DP can affect coverage of the surrounding CTV. Conformity might improve by extending heterogeneous prescriptions to the CTV margins or in patients with larger GTVs, as in our study the GTV was substantially smaller than the CTV (Table S1).

The DP approach allowed dose escalation within target regions without increasing OAR doses, aligning with previous findings in proton therapy. Köthe et al. demonstrated dose escalation to hypoxic regions in non-small cell lung cancer did not increase normal tissue toxicity [19], while Wang et al. reported similar findings in intraprostatic lesions [20]. Whether higher target doses translate into survival benefits, such as improved local control or reduced neurological symptoms, remains to be determined. We observed median D_max_ values higher in the CTV than in the GTV (Tables S5 and S6) due to hotspots in safety margins (Fig. S1), reflecting limitations of PBS with fixed beams compared with photon VMAT or IMRT techniques for delivering heterogeneous doses while sparing surrounding tissues.

TCP estimation using the Poissonian LQ model suggested DP could improve local control within the GTV versus uniform plans. However, estimated TCP values differed from the observed 5-year local control (84 %) in our SBC cohort [24] and other proton studies [[36], [37], [38]]. This result likely reflects differences in TPS re-optimisation and our use of a fixed, escalated prescription dose of 74 Gy(RBE) to the high-risk target, consistent with our clinical practice [24]. We did not include sequential or randomised boost arms to avoid confounding complexity in this technical feasibility study. Future research should compare biologically guided DP to spatially agnostic boosts, explore larger cohorts, and test alternative TCP models and cellularity estimations.

Limitations of our study include assumptions in the TCP model, which used uniform α and β values derived from photon radiotherapy studies, ignoring intratumoral radiosensitivity heterogeneity and potentially leading to lower TCP estimates than those clinically observed [24]. Cellular-based dose prescriptions relied on a microstructural model with simplifying assumptions—spherical/ellipsoidal cells, all clonogenic, and unvalidated for SBC [[39], [40], [41]]—potentially underestimating TCP. The modest TCP gains observed likely reflect limitations of the linear cellularity-to-dose mapping and restriction of DP to the GTV, leaving few voxels differing from uniform plans.

Despite these constraints, our linear mapping method prioritised reproducibility and alignment with prior DP studies [12], demonstrating the feasibility of DWI-guided DP in a clinical TPS rather than validating ADC-derived cellularity for outcome-driven escalation. DP may yield greater TCP gains in tumours where the GTV represents a larger portion of the CTV or if prescriptions are extended to the CTV. Preliminary analyses exploring patient-specific cellularity-to-dose mapping extended to the CTV margins highlight potential (Supplementary C), but this strategy requires refinement due to inaccuracies in cellularity estimation in bone-dense CTV margins.

This work should be viewed as a feasibility-oriented technical note rather than a full biological validation study. Within the spectrum of dose painting strategies, contour-based or multi-level escalation using anatomical or functional imaging has been clinically explored [42], whereas voxel-based biological optimisation directly embedding TCP models into the planning process represents a more advanced direction [43]. Our contribution lies between these approaches: by demonstrating voxel-level inverse prescription mapping in a commercial proton TPS, we provide a practical, reproducible implementation pathway. This intermediate positioning highlights technical feasibility while acknowledging that further biological validation and integrated optimisation are necessary before clinical translation.

Finally, while demonstrated in proton therapy, this approach could be applied to carbon ion therapy in future studies. Overall, DP proton therapy is technically feasible, enabling higher target doses without increasing OARs exposure, warranting further investigation with larger cohorts, diverse tumour sites, and refined DP and TCP strategies to optimise local control and survival outcomes.

## Declaration of competing interest

The authors declare that they have no known competing financial interests or personal relationships that could have appeared to influence the work reported in this paper.

## Footnotes

1 Gy(RBE) refers to the ICRU93 definition.
