# Supplementary material for: Evaluating the technical feasibility of biology-guided dose painting in proton therapy
Source: Phys Imaging Radiat Oncol. 2025 Aug 27;35:100832. doi: 10.1016/j.phro.2025.100832 (PMC12414908; doi:10.1016/j.phro.2025.100832)
Supplement: Supplementary Data 1 [file mmc1.pdf]

Supplementary Information

Supplementary tables

Table S1. Clinical characteristics of skull-base chordoma patients included in this study.

Patient #	Sex	Age (years)	Time between pretreatment MRI and panning CT (days)	Time between pretreatment MRI and start of radiotherapy (days)	GTV volume [cm^3]	CTV volume [cm^3]	Local control [yes=0; no=1]	Follow-up time [months]	Local relapse time [months]
1	F	58	0	19	11.1	26.0	0	89.6	-
2	M	63	1	14	0.8	6.8	1	53.2	40.5
3	F	64	1	13	1.3	30.3	0	85.4	-
4	F	37	0	14	0.4	9.4	1	56.9	18.0
5	M	42	0	21	9.3	23.2	1	76.3	21.2
6	M	60	0	22	3.9	45.7	0	76.0	-
7	F	45	0	17	14.0	53.0	1	49.0	33.7
8	M	65	0	20	8.2	26.8	1	51.5	58.2
9	M	70	0	14	1.6	57.3	1	41.4	29.1
10	M	40	0	20	11.8	118.8	0	50.4	-

Table S2. Clinical goals set for radiotherapy targets and organs at risk.

Region of interest	Dose painting plans	Uniform plans
GTV	at least 70.3 Gy(RBE) at 95% volume	
GTV	at most 83.5 Gy(RBE) at 1% volume	at most 76.5Gy (RBE) at 1% volume
CTV	at least 70.3 Gy(RBE) at 95% volume	
CTV	at most 83.5 Gy(RBE) at 1% volume	at most 76.5 Gy(RBE) at 1% volume
Brainstem	at most 63 Gy(RBE) at 0.01 cm^3 volume	
Chiasm	at most 54 Gy(RBE) at 1% volume	
Optic nerves	at most 54 Gy(RBE) at 1% volume	
Cochleae	at most 45 Gy(RBE) average dose	
Carotids	at most 75.5 Gy(RBE) at 1% volume	
Temporal lobes	at most 71 Gy(RBE) at 2 cm^3 volume	

Table S3. Values of local control, mean cellularity, D_{max}, $D_{95\%}$,$D_{1\%}$, tumour control probability and mean quality factor in the gross tumour volume obtained with dose painting.*

Patient #	LC	Mean cellularity [10^4]	D_{max} [Gy(RBE)]	$D_{95\%}$ [Gy(RBE)]	$D_{1\%}$ [Gy(RBE)]	TCP [0-1]	QF [%]
**1**	1	2102	78.4	72.9	76.8	0.00	98.9
**2**	0	1814	79.2	71.9	77.8	0.68	97.8
**3**	1	2038	78.8	73.1	77.4	0.72	99.0
**4**	1	1107	76.7	73.2	76.0	0.87	98.4
**5**	0	2127	81.7	66.5	79.3	0.00	97.2
**6**	1	3112	77.8	73.3	77.1	0.02	98.6
**7**	0	567	78.0	71.7	76.4	0.03	98.5
**8**	1	564	77.9	72.5	76.4	0.18	98.8
**9**	0	895	77.7	72.5	76.2	0.70	98.5
**10**	1	460	79.5	72.4	76.4	0.09	98.8

Table S4. Values of local control, mean cellularity, D_{max}, $D_{95\%}$,$D_{1\%}$, tumour control probability and mean quality factor in the gross tumour volume obtained with uniform prescription plans. Values of mean cellularity are the same as those reported in Table S3 as they were also used to calculate TCP with the dose delivered by the uniform prescription plans.*

Patient #	LC	Mean cellularity [10^4]	D_{max} [Gy(RBE)]	$D_{95\%}$ [Gy(RBE)]	$D_{1\%}$ [Gy(RBE)]	TCP [0-1]	QF [%]
**1**	1	2102	75.9	72.9	74.4	0.00	99.5
**2**	0	1814	76.9	71.2	76.2	0.62	98.1
**3**	1	2038	75.2	72.5	74.5	0.65	99.5
**4**	1	1107	75.3	73.3	74.6	0.81	99.5
**5**	0	2127	77.0	65.6	75.5	0.00	97.9
**6**	1	3112	75.8	72.9	75.1	0.004	99.3
**7**	0	567	76.4	70.3	75.7	0.01	98.9
**8**	1	564	76.3	72.4	74.7	0.12	99.4
**9**	0	895	76.8	72.4	75.3	0.65	99.1
**10**	1	460	78.7	72.5	75.6	0.07	99.2

Table S5. Results of the statistical analysis comparing dose metrics in the gross tumour volume between the dose painting and the uniform plans. Values represent median (range).†

Dose metric	Dose painting [Gy(RBE)]	Uniform [Gy(RBE)]	p value
D_{mean}	74.9 (74.7-75.5)	74.3 (73.6-74.8)	0.01
D_{median}	75.1 (74.6-75.7)	74.3 (74.1-75.4)	0.01
D_{max}	78.2 (76.7-81.7)	76.4 (75.2-78.7)	0.01
D_{min}	69.1 (51.8-73.5)	68.3 (53.0-73.7)	0.3
$D_{95\%}$	72.5 (66.5-73.3)	72.5 (65.6-73.3)	0.05
$D_{1\%}$	76.6 (76.0-79.3)	75.2 (74.4-76.2)	0.01

* D_{max}, maximum dose; $D_{95\%}$, dose to 95% of volume; $D_{1\%}$, dose to 1% of volume; LC, local control; TCP, tumour control probability; QF, quality factor.

† D_{mean}, mean dose; D_{median}, median dose; D_{max}, maximum dose; D_{min}, minimum dose; $D_{95\%}$, dose to 95% of volume; $D_{1\%}$, dose to 1% of volume. N=10.

Table S6. Results of the statistical analysis comparing dose metrics in the clinical target volume between the dose painting and the uniform plans. Values represent median (range).‡

Dose metric	Dose painting [Gy(RBE)]	Uniform [Gy(RBE)]	p value
D_{mean}	74.6 (74.1-75.0)	74.1 (73.5-74.3)	0.01
D_{median}	74.6 (74.2-75.3)	74.2 (74.1-74.4)	0.01
D_{max}	79.0 (78.0-81.7)	76.7 (75.9-78.7)	0.01
D_{min}	63.6 (51.6-70.6)	61.5 (52.5-70.5)	0.6
$D_{95\%}$	72.1 (66.0-73.0)	72.0 (65.6-72.7)	0.1
$D_{1\%}$	76.4 (76.1-78.5)	75.1 (74.4-76.1)	0.01

Table S7. Results of the statistical analysis comparing clinical goals in the organs at risks between the dose painting and the uniform plans. Values represent median (range).‡

Clinical goal	Dose painting [Gy(RBE)]	Uniform [Gy(RBE)]	p value
Brainstem D_{max} 0.01 cm^3	59.8 (57.4-61.2)	60.6 (59.6-61.7)	0.03
Chiasm $D_{1\%}$	48.3 (26.3-50.6)	47.0 (28.7-50.4)	0.8
Optic nerve R $D_{1\%}$	46.5 (6.0-52.5)	47.3 (10.0-53.0)	0.1
Optic nerve L $D_{1\%}$	32.6 (9.7-54.0)	34.8 (15.1-52.5)	0.4
Carotid R $D_{1\%}$	75.7 (75.2-76.6)	74.7 (73.4-76.1)	0.03
Cochlea R D_{mean}	32.7 (7.6-45.0)	35.4 (7.5-45.2)	0.08
Cochlea L D_{mean}	40.0 (6.5-45.1)	43.0 (9.0-45.2)	0.4
Temp Lobe R D_{max} - 2 cm^3	41.5 (32.8-64.5)	47.1 (32.5-67.3)	0.03
Temp Lobe L D_{max} - 2 cm^3	43.0 (31.0-74.3)	46.2 (31.3-73.7)	0.1

‡ D_{mean}, mean dose; D_{median}, median dose; D_{max}, maximum dose; D_{min}, minimum dose; $D_{95\%}$, dose to 95% of volume; $D_{1\%}$, dose to 1% of volume. N=10.

Table S8. Estimates of Equivalent Uniform Dose in units of Gy(RBE) for organs at risk obtained with dose painting.§

Patient #	Brainstem	Chiasm	Optic nerve L	Optic nerve R	Cochlea L	Cochlea R	Temporal lobe L	Temporal lobe R
1	32.5	41.1	30.9	31.1	25.4	23.6	33.0	35.2
2	34.8	36.2	0.6	0.6	7.9	8.1	31.4	29.4
3	34.8	35.9	12.7	6.9	41.0	32.7	31.4	23.2
4	31.9	39.2	34.5	32.3	14.4	11.3	28.6	35.0
5	33.6	41.1	31.8	31.7	22.6	0.7	56.5	31.1
6	38.7	41.3	34.3	37.3	42.5	34.7	36.8	38.4
7	37.8	27.5	15.5	19.4	46.2	41.4	24.8	28.7
8	35.6	20.6	6.7	3.2	46.0	34.9	21.8	22.7
9	35.0	21.3	0.6	0.6	39.6	27.7	25.6	24.0
10	38.7	37.8	2.7	0.9	43.5	35.8	44.3	35.2

Table S9. Estimates of Equivalent Uniform Dose in units of Gy(RBE) for organs at risk obtained with uniform prescription plans.§

Patient #	Brainstem	Chiasm	Optic nerve L	Optic nerve R	Cochlea L	Cochlea R	Temporal lobe L	Temporal lobe R
1	34.9	41.3	31.0	31.8	25.7	23.4	33.1	35.6
2	34.7	35.8	0.6	0.6	11.3	10.4	32.8	31.8
3	37.0	36.0	15.8	9.1	42.4	34.6	31.3	23.5
4	32.4	38.4	32.9	32.0	12.8	11.3	30.5	35.9
5	33.4	41.9	32.4	32.6	22.6	0.7	56.1	31.3
6	39.5	37.8	34.5	36.6	44.1	39.9	38.1	39.8
7	38.3	29.3	18.3	22.3	45.9	41.9	25.8	30.9
8	37.2	22.8	9.4	5.5	46.3	37.4	21.5	24.3
9	37.3	23.5	0.6	0.6	44.4	30.2	27.0	24.6
10	38.0	37.4	4.4	3.5	43.5	36.6	43.5	35.4

§ As per Hall et al. Int J Radiation Oncol Biol Phys,Vol.97, No.5, pp.1087-1094, 2017, the tissue-specific parameter a describing the volume effect was taken to be 7 for the brainstem, 25 for the optic chiasm and optic nerves, 1 for the cochlea and 5 for the temporal lobes.

Supplementary figures

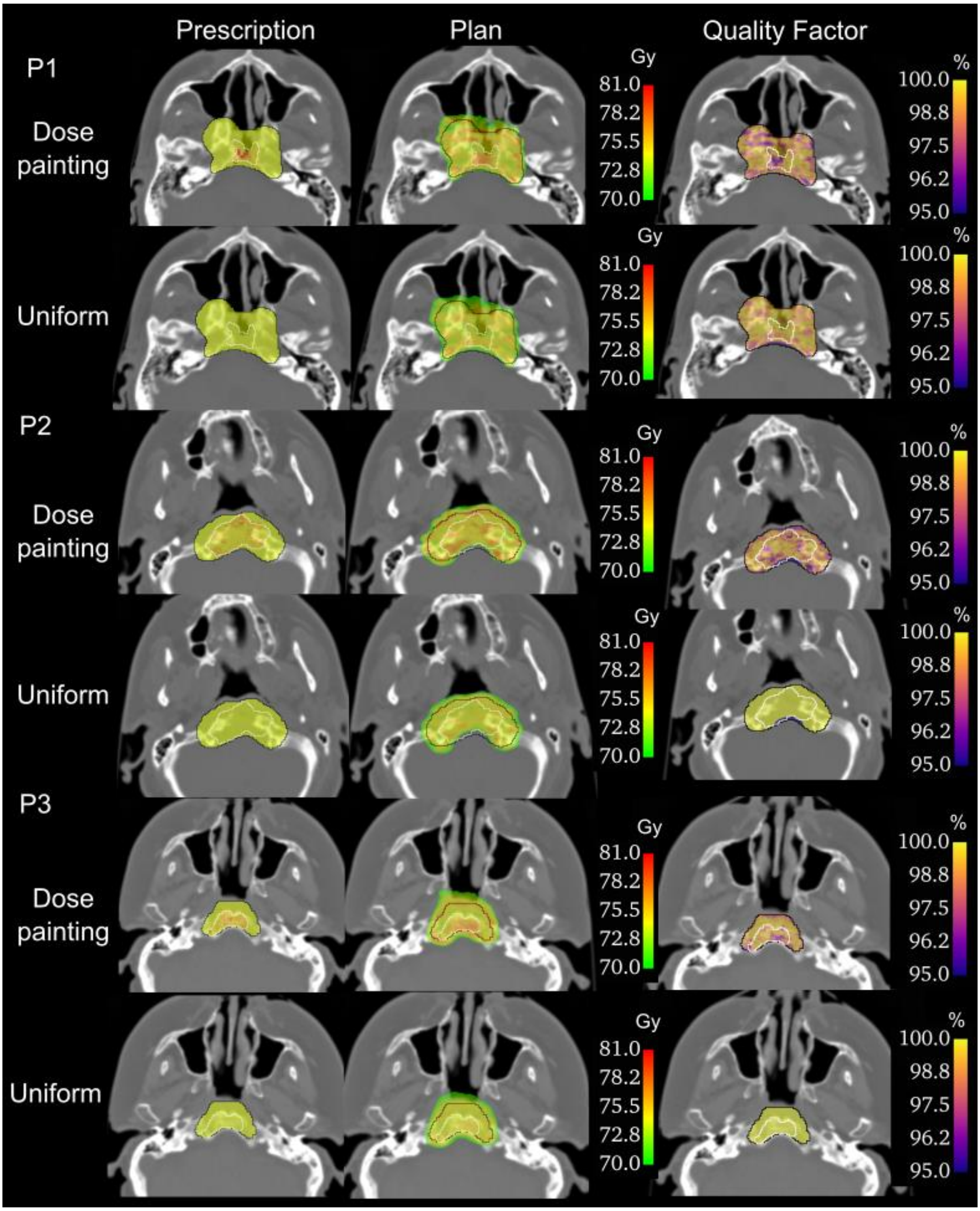

Figure S1. *Examples of CT scans with overlayed dose prescriptions, dose plans and quality factor maps calculated within the clinical target volume for four skull-based chordoma patients*. The black contour indicates the clinical target volume (CTV) and the white contour indicated the gross tumour volume (GTV).

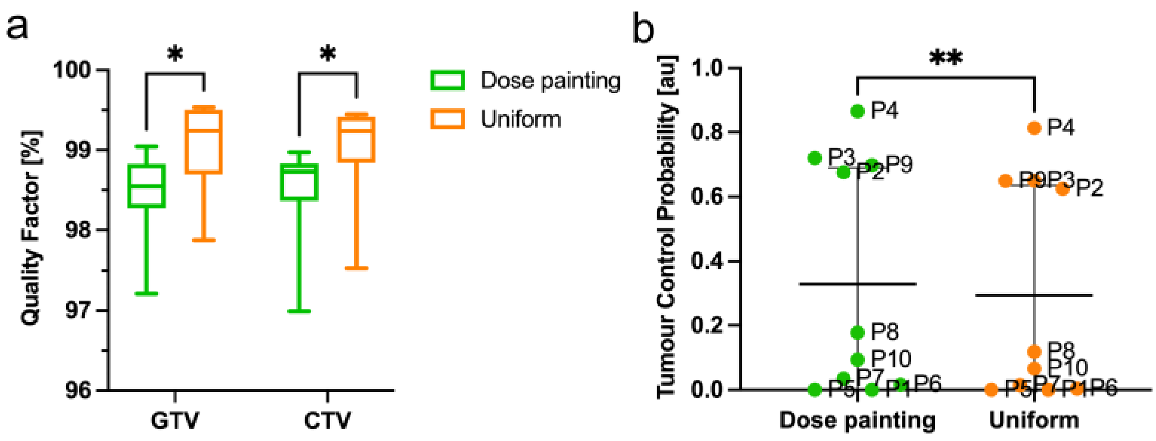

Figure S2. *Plans conformity and local tumour control probability estimates*. **a**) Box and whiskers plot of comparison of quality factor between dose painting and uniform plans in the GTV and CTV. The bar in the boxes indicates the median value. **b**) Plot of comparison of tumour control probability between dose painting and uniform plans in the GTV. The bar indicates the group mean and the whiskers indicate the standard deviation. CTV, clinical target volume; GTV, gross tumour volume. *$p < 0.05$, **$p < 0.01$. N=10.

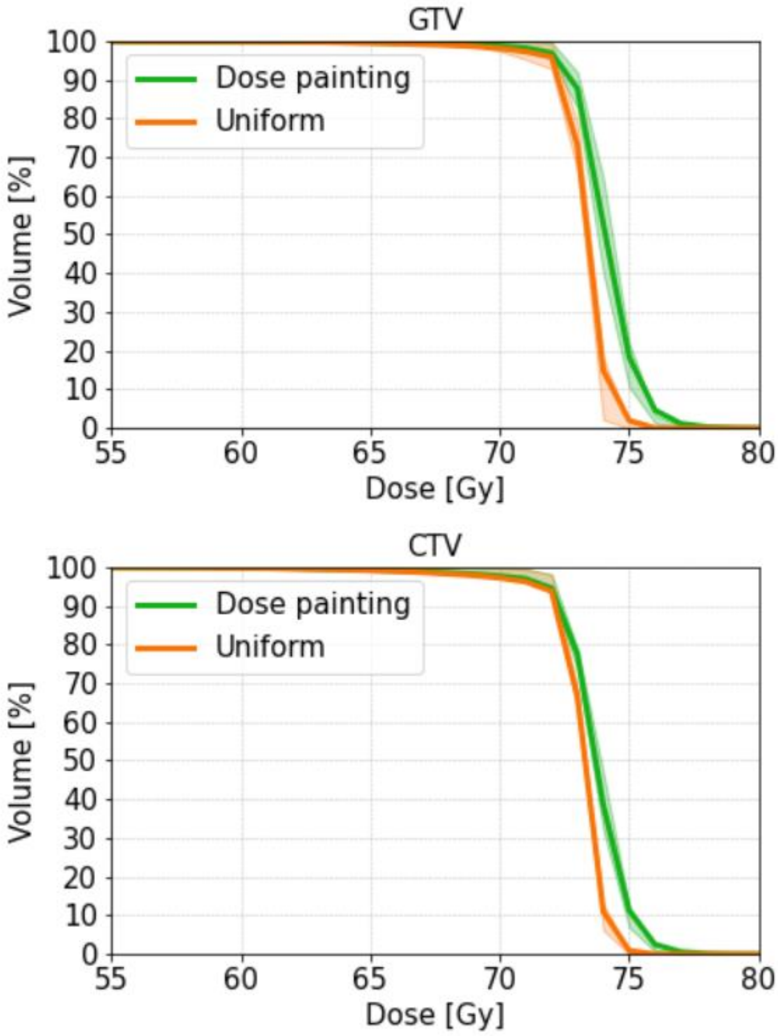

Figure S3. *Dose volume histograms of target volumes*. Mean dose volume histograms for the GTV and CTV for the whole cohort of patients (solid lines) are shown for dose painting (green) and uniform (orange) proton plans. Colour bands represent the cohort 25th to 75th percentile. CTV, clinical target volume; GTV, gross tumour volume.

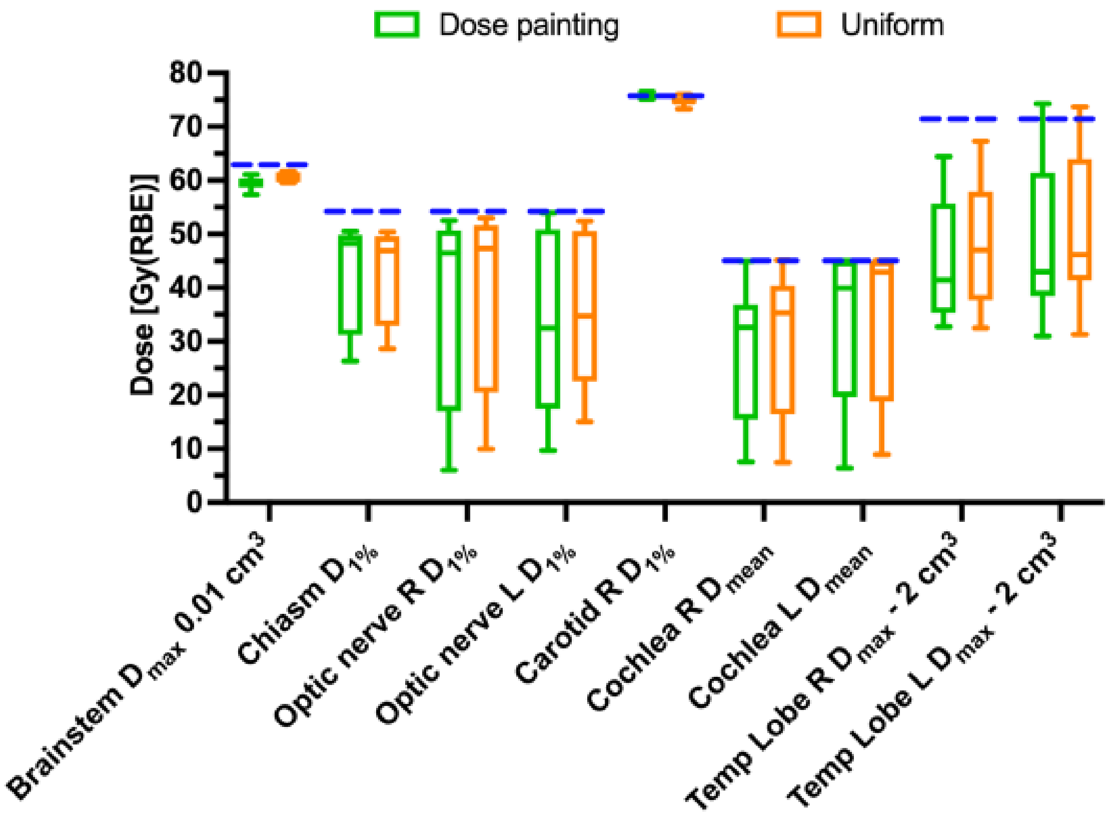

Figure S4. *Dose metrics to organs at risk*. Box and whiskers plot of comparison of dose metrics in the organs at risk between dose painting and uniform plans. The bar in the boxes indicates the median value. D_{mean}, mean dose; D_{max}, maximum dose; $D_{1\%}$, dose to 1% of volume; L, left; R, right. Blue dotted lines represent the clinical goal values for each organ at risk. All comparisons are not significant, $p > 0.05$. N=10.

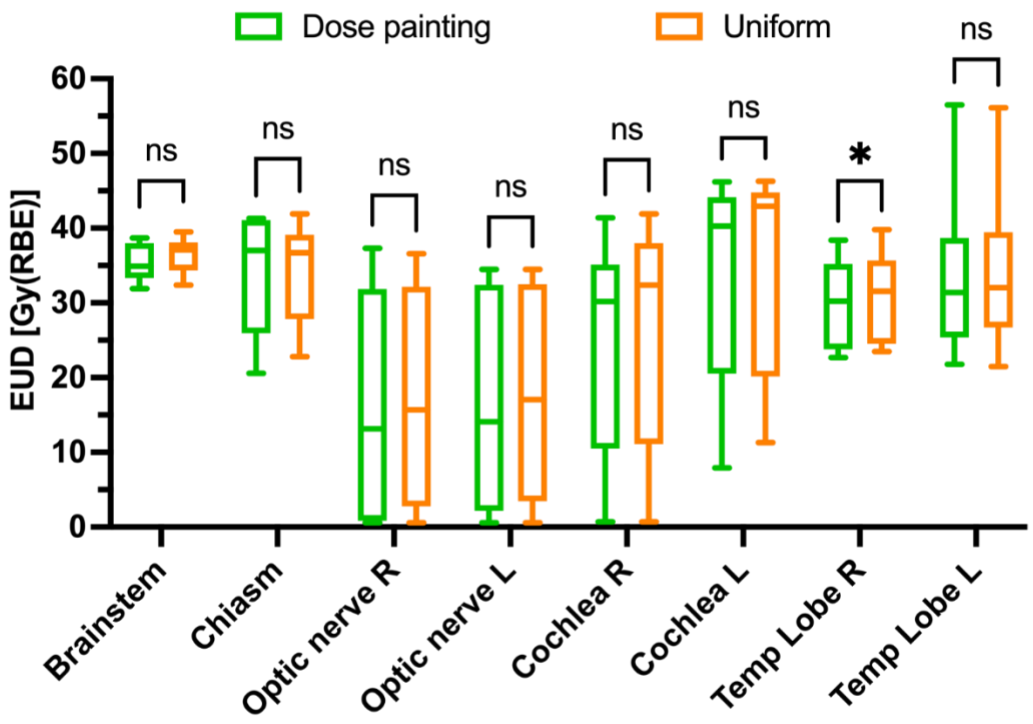

Figure S5. *Equivalent uniform dose estimates in organs at risk*. Box and whiskers plot of comparison of equivalent uniform dose in the organs at risk between dose painting and uniform plans. The bar in the boxes indicates the median value. EUD, equivalent uniform dose; L, left; R, right. *$p < 0.05$, ns = $p > 0.05$. N=10.[**]

[**] As per Hall et al. Int J Radiation Oncol Biol Phys,Vol.97, No.5, pp.1087-1094, 2017, the tissue-specific parameter a describing the volume effect was taken to be 7 for the brainstem, 25 for the optic chiasm and optic nerves, 1 for the cochlea and 5 for the temporal lobes.

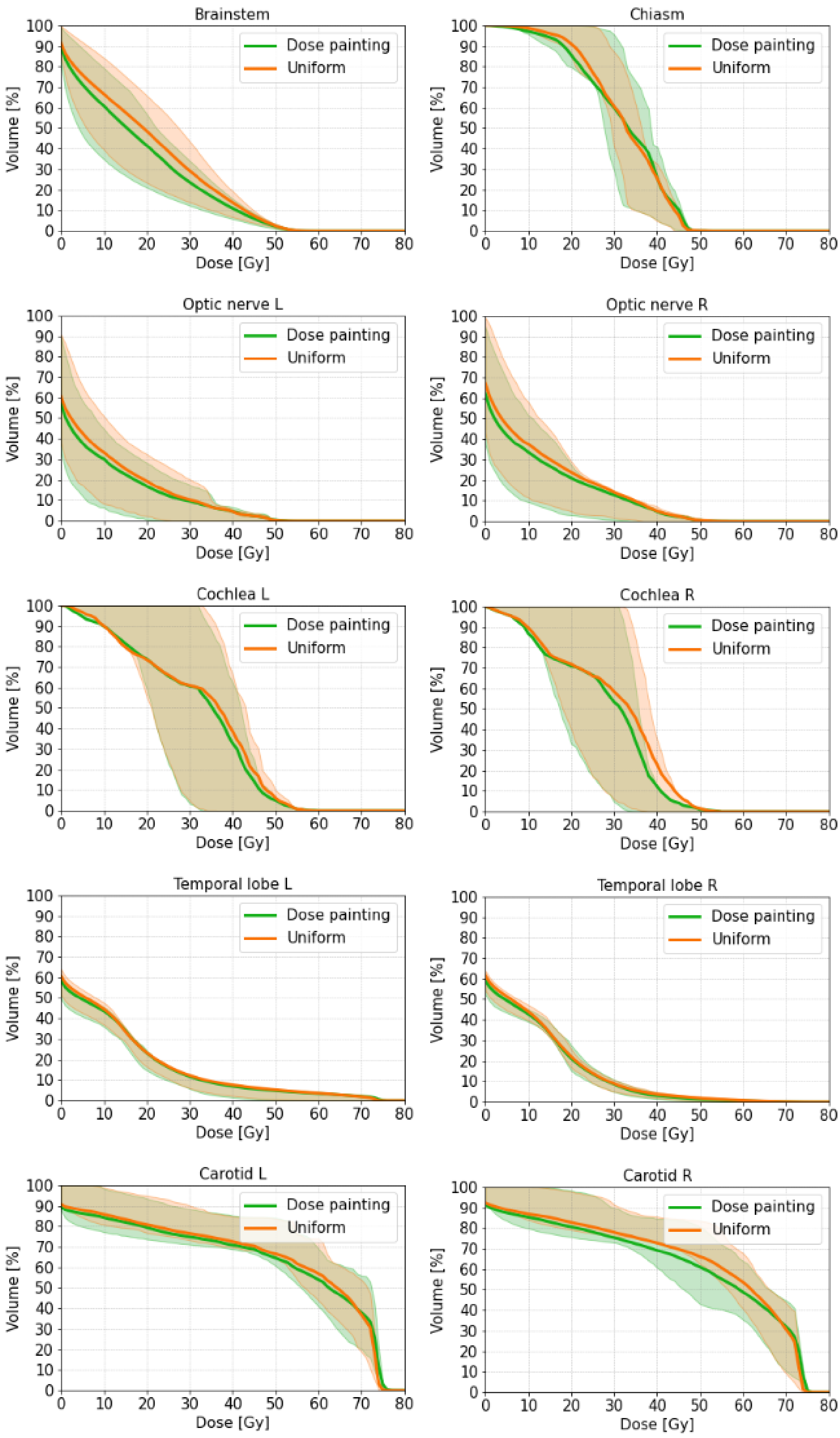

Figure S6. *Dose volume histograms of organs at risk*. Mean dose volume histograms for the organs at risk for the whole cohort of patients (solid lines) are shown for dose painting (green) and uniform (orange) proton plans. Colour bands represent the cohort 25th to 75th percentile. L, left; R, right.

Supplementary A

1. Patient dataset

This study used anonymised retrospective data from ten SBC patients treated with proton therapy at the National Center for Oncological Hadrontherapy (CNAO) between 2013 and 2017. All patients received surgery and had macroscopical residual tumour at the pretreatment MRI assessment. The data were retrospectively collected within the scope of the AIRC project (Ref. 24946) approved by the CNAO ethics committee (CNAO 43 2021). For each patient, T_2-weighted and DWI images, acquired prior to radiotherapy, were collected, along with radiotherapy planning data, including CT images, target volumes and organs at risk (OARs) contours. The median and range of days lapsed between the pretreatment MRI and the planning CT was 0 (0-1) days, and between pre-treatment MRI and treatment start was 18 (13-22) days (**Table S1**). Radiotherapy targets included the gross tumour volume (GTV), contoured on planning CT with T_2-weighted MRI overlay, and the clinical target volume (CTV), defined as the GTV plus a 3–5 mm margin adjusted for anatomy and the surgical pathway. Inclusion criteria required DWI-MRI data acquired with a 32-channel head coil on a 3T scanner (Magnetom Verio Siemens) with the following acquisition parameters: TE = 66–83 ms, TR = 5600–6600 ms, flip angle = 90°; GRAPPA factor = 2, resolution = 1.98×1.98×5 mm, and b values of 50, 400 and 1000 s/mm2. Patients' characteristics are provided in **Table S1**.

2. Image processing

DWI data were used to derive apparent diffusion coefficient (ADC) maps with a mono-exponential fit of the DWI signal at b values 50, 400, 1000 s/mm2. Cellular density maps were derived from ADC maps using a previously developed model [1–3]. Cellular density estimates were derived only within the GTV, assuming the DWI-cellularity model applies only to voxels with certain residual tumour. Radiotherapy planning data were co-registered to cellularity maps by linear rigid registration of the CT image to the T2-weighted image, followed by resampling to the DWI image resolution using SimpleITK [4].

Supplementary B

1. Dose painting plans optimisation and quality assessment

All the objectives were set for the beamset dose, except for the objective of delivering a uniform dose of 81 Gy(RBE) to the CTV, which was applied for the optimisation of the beamset combined with the background dose. Following optimisation, a robust evaluation on the beamset effective dose was conducted using 2 mm setup margins and 3% range uncertainty. The assessment verified whether the nominal dose to OARs, the percentage scenarios in which the clinical goals were met, and the worst-case scenario were clinically acceptable, as determined by an experienced medical physicist. If required, objective weightings were adjusted and the plan was re-optimised until clinically acceptable by robust evaluation.

2. Plan comparison strategy

To enable a fair comparison between dose painting (DP) and conventional uniform plans, we opted to deliver a uniform prescription dose of 74 Gy(RBE) in 37 equi-dose fractions to the high-risk (HR) volume for both plan types. This dose level aligns with our institutional protocol, which typically involves a sequential boost: 54 Gy(RBE) to the low-risk (LoR) volume followed by a 20 Gy(RBE) boost to the HR volume [5]. However, implementing a sequential boost strategy within this study would have introduced unnecessary complexity and potential confounding in the optimisation process,

especially given the aim of isolating the technical feasibility of DWI–guided heterogeneous dose prescription. As such, we re-planned both conventional and DP plans with a single-phase optimisation, maintaining identical target volumes and fractionation schedules across both arms. This approach ensured that any differences observed between the two plans were attributable to the dose distribution strategy (homogeneous vs. heterogeneous), rather than differences in total prescribed dose or boost technique.

Supplementary C

1. **Patient-specific cellularity-to-dose mapping strategies**

The following formulations were developed by making use of the radiotherapy planning data originally used for the treatment of the SBC patients included in this study. Specifically, as described in *Iannalfi et al.* [5] "Patients were treated according to a sequential boost protocol. The prescription dose was 74 Gy(RBE) delivered in 37 fractions of 2 Gy(RBE): 54 Gy(RBE) to the low risk (LoR) volume and 20 Gy(RBE) to the high risk (HR) volume. The HR clinical target volume (CTV-HR) included GTV with 3–5 mm safety margin modified according to both the anatomy and the surgical pathway, to include the HR areas of tumor recurrence. In case of macroscopically radical resection, the CTV-HR was limited to include the resection margins. The CTV-LoR was obtained by adding 5 mm (isotropic expansion) to CTV-HR, then it was modified according to preoperative extension of disease, surgical pathway, and postoperative changes. The HR planning target volume (PTV) and PTV-LoR encompassed the CTV-HR and CTV-LoR, respectively with a uniform three-dimensional margin of 2 mm."

3.1. Option 1

This approach consists in mapping the dose based on the cellular density distribution only within the GTV.

<u>Assumptions</u>: tumour with higher cellular density requires a higher dose to achieve higher TCP [6]. Thus, the per-voxel dose prescription assignment should be scaled either following the shape of the cumulative density function of the cellular density or linearly within the GTV.

<u>Method</u>

Mapping the dose according to cellular density in the GTV could be done in the following ways:

a) setting D_{min}=$D_{99th\ perc}$, $D_{N_{mode}}$= 74 Gy(RBE), and scaling the dose according to the cdf of the cellular density distribution within the GTV;
b) setting D_{min}=$D_{99th\ perc}$, $D_{N_{max}}$= 110% of $D_{99th\ perc}$, and scaling the dose linearly with respect to the cellular density within the GTV;
c) setting D_{min}=$D_{99th\ perc}$, D_{mean} in the GTV = 74 Gy(RBE), and scaling the dose linearly with respect to the cellular density within the GTV.

<u>Problem</u>: In some cases D_{min} was < 74 Gy(RBE), which is the clinical standard of care. The GTV should NOT be prescribed a dose below clinical standard of care, as this would result in worse patient outcome. Therefore, D_{min} should be set to 74 Gy(RBE). For options a) and c) the D_{presc} to the voxels with the highest cellular density was significantly higher than what is clinically acceptable. Therefore, the D_{presc} to the voxels with the highest cellular density should be capped at the highest clinically acceptable dose, which is 110% of dose delivered to HR volume as per standard of care (i.e. 81 Gy(RBE)).

<u>Outcome</u>: Perform dose mapping only within the GTV, set D_{min}=74 Gy(RBE) and D_{max}=81 Gy(RBE), and scaling linearly in between.

3.2. Option 2

The second mapping strategy we tested consisted in scaling the dose distribution following the shape of the cumulative density function of the cellular density within the CTV-LoR, as described below.

Assumptions: tumour with higher cellular density requires a higher dose to achieve higher TCP.[6] Thus, the per-voxel dose prescription assignment should be scaled following the shape of the cumulative density function of the cellular density within the CTV.

The ADC-cellularity model was developed from simulations on SBC tumour tissue substrates within a limited range of ADC values. While this range covers the ADC signals measured from most voxels of SBC patients, a small percentage of signals deriving from voxels involving bone structures is incompatible with the simulations. Consequently, the cellularity estimates in voxels involving bone structures is unreliable and cannot be used to derive a dose prescription.

Method

Firstly, we derived the number of cells per voxels within the CTV-LoR to calculate a patient-specific tumour control probability (TCP) in the dose range 0-100 Gy, according to the Poissonian LQ model:

$$TCP = \prod_{i=1}^{P} e^{-N_i e^{\left(-\alpha_{ion} D_i - \frac{D_i^2 \beta_{ion}}{n}\right)}} \quad (1)$$

where N_i is the number of cells per voxel, D_i is the dose delivered per voxel, n is the number of fractions corresponding to 37 and P is the total number of voxels within the CTV-LoR. The dose corresponding to the 99th percentile of the TCP distribution ($D_{99th\ perc}$) was recorded. For each patient, the probability density distribution of N_i was derived and a kernel density estimate (kde) of N_i was obtained using Gaussian kernels.

The cumulative density function of the kde of N_i (cdf_kde) was computed by integrating the kde over the range of values of N_i. The values of N_i were converted in dose prescriptions ($D_{presc,i}$) based on the shape of the cdf_kde, according to the following formulae:

$$D_{presc,i} = \left(D_{99th\ perc} - D_{min}\right) \cdot \frac{cdf_{kde(N_i)}}{cdf_{kde(N_{mode})}} + D_{min} \quad (2)$$

where D_{min} is the minimum prescribed dose corresponding to the clinical standard (i.e. 54 Gy(RBE)), $cdf_{kde(N_i)}$ is the value of cdf_kde calculated for N_i, N_{mode} is the mode of the distribution and $cdf_{kde(N_{mode})}$ is the value of cdf_kde calculated for N_{mode}. The choice of assigning a dose prescription of D_{99th} to voxels with the highest probability density of cellularity (N_{mode}) in the CTV-LoR stemmed from the goal to optimise the TCP=1 within the CTV-LoR. As TCP is calculated from the product of all voxels (Eq. 1), the highest fraction of voxels with a particular cellularity (N_{mode}) should weight more on the overall TCP calculation.

The $D_{presc,i}$ was then capped to 110% of the dose delivered to HR target volume as per the original boost plans (i.e. 81.4 Gy(RBE)). For voxels within the CTV-HR for which values of cellularity were not reliable or that involved bone structures, a $D_{presc,i}$ of 74 Gy(RBE) was assigned to reflect the dose prescribed to the CTV-HR in the boost plans. For voxels outside the CTV-HR but within the CTV-LoR for which values of cellularity were not reliable or that involved bone structures, a $D_{presc,i}$ of 54 Gy was assigned to reflect the dose prescribed to the CTV-LoR in the boost plans.

Problems: some voxels within the GTV receive a lower prescription than in the original boost plans, and, consequently, they received a lower dose in the plan. From a clinical standpoint, it is not acceptable to underdose the GTV, which is, by definition, leftover tumour.

Additionally, many voxels within the CTV margins of expansion of SBC patients involve bone structures and therefore receive a standard dose rather than a heterogeneous dose prescription.

Outcome: this approach was excluded as the cellularity-to-dose mapping formulation needs further refinement, and the prevalence of bone structures in the CTV margins limits the application of this DP approach.

References

[1] Morelli L, Buizza G, Paganelli C, Riva G, Fontana G, Imparato S, et al. A Microstructure Model from Conventional Diffusion MRI of Meningiomas: Impact of Noise and Error Minimization. In: Cetin-Karayumak S, Christiaens D, Figini M, Guevara P, Gyori N, Nath V, et al., editors. Computational Diffusion MRI, Cham: Springer International Publishing; 2021, p. 25–35. https://doi.org/10.1007/978-3-030-87615-9_3.

[2] Morelli L, Palombo M, Buizza G, Riva G, Pella A, Fontana G, et al. Microstructural parameters from DW-MRI for tumour characterization and local recurrence prediction in particle therapy of skull-base chordoma. Med Phys 2023:1–14. https://doi.org/10.1002/mp.16202.

[3] Buizza G, Paganelli C, Ballati F, Sacco S, Preda L, Iannalfi A, et al. Improving the characterization of meningioma microstructure in proton therapy from conventional apparent diffusion coefficient measurements using Monte Carlo simulations of diffusion MRI. Med Phys 2021;48:1250–61. https://doi.org/https://doi.org/10.1002/mp.14689.

[4] Lowekamp BC, Chen DT, Ibáñez L, Blezek D. The design of simpleITK. Front Neuroinform 2013;7:1–14. https://doi.org/10.3389/fninf.2013.00045.

[5] Iannalfi A, D'Ippolito E, Riva G, Molinelli S, Gandini S, Viselner G, et al. Proton and carbon ion radiotherapy in skull base chordomas: a prospective study based on a dual particle and a patient-customized treatment strategy. Neuro Oncol 2020;22:1348–58. https://doi.org/10.1093/neuonc/noaa067.

[6] Webb S, Nahum AE. A model for calculating tumour control probability in radiotherapy including the effects of inhomogeneous distributions of dose and clonogenic cell density. Phys Med Biol 1993;38:653–66. https://doi.org/10.1088/0031-9155/38/6/001.
